# Simultaneous giant mucinous cystadenoma of the appendix and intestinal schistosomiasis: ‘case report and brief review’

**DOI:** 10.1186/1477-7819-12-385

**Published:** 2014-12-17

**Authors:** Changwei Lin, Xiaorong Li, Yihang Guo, Gui Hu, Yi Zhang, Kaiyan Yang, Yi Gan, Jianyu Zhou, Lv Lv, Kai Gao, Juan Du

**Affiliations:** Department of General surgery, The Third XiangYa Hospital of Central South University, Tongzipo Road, Changsha, 410013 Hunan, PR China

**Keywords:** appendix, mucinous cystadenoma, schistosomiasis

## Abstract

Both mucinous cystadenoma of the appendix and intestinal schistosomiasis are rare lesions. We report a rare case of simultaneous giant mucinous cystadenoma of the appendix and intestinal schistosomiasis. A 64-year-old man from China presented with a one-year history of pain in the right lower quadrant of the abdomen. There were no other pertinent historical findings, other than schistosomiasis. Imaging showed a large, tubular, mesenteric cystic structure extending downwards from the inferior wall of the cecum. Right hemicolectomy was performed for the appendiceal tumor. The final pathological diagnosis was mucinous cystadenoma with calcified Schistosome eggs within the mucosa and submucosa of the appendix, small intestine, colon, and lymph nodes. We deduced that the pathogenesis of appendiceal mucinous cystadenoma in our case was Schistosome eggs causing luminal obstruction, finally resulting in intraluminal accumulation of mucoid material. Postoperatively, the patient recovered well.

## Background

A mucocele is an uncommon pathology of the appendix, characterized by an obstructive dilatation of the appendix caused by intraluminal accumulation of mucoid material. The incidence is observed in 0.2% to 0.7% of all appendectomied specimens [1, 2]. According to the modern classification, four subgroups of the disease can be identified: simple mucocele, mucosal hyperplasia, mucinous cystadenoma, and mucinous cystadenocarcinoma [3]. Mucoceles, associated with mucoid material in the peritoneal cavity, are caused by mucus distention secondary to an obstruction of the appendix due to fecaliths, post-inflammatory scarring, or, rarely, Schistosome eggs. There are only three previously reported cases concerning mucinous cystadenoma of the appendix and intestinal schistosomiasis in the English literature [4–6]. Here, we present a rare case of simultaneous giant mucinous cystadenoma of the appendix and intestinal schistosomiasis and provide a brief review of the literature.

## Case presentation

**Figure 1:**
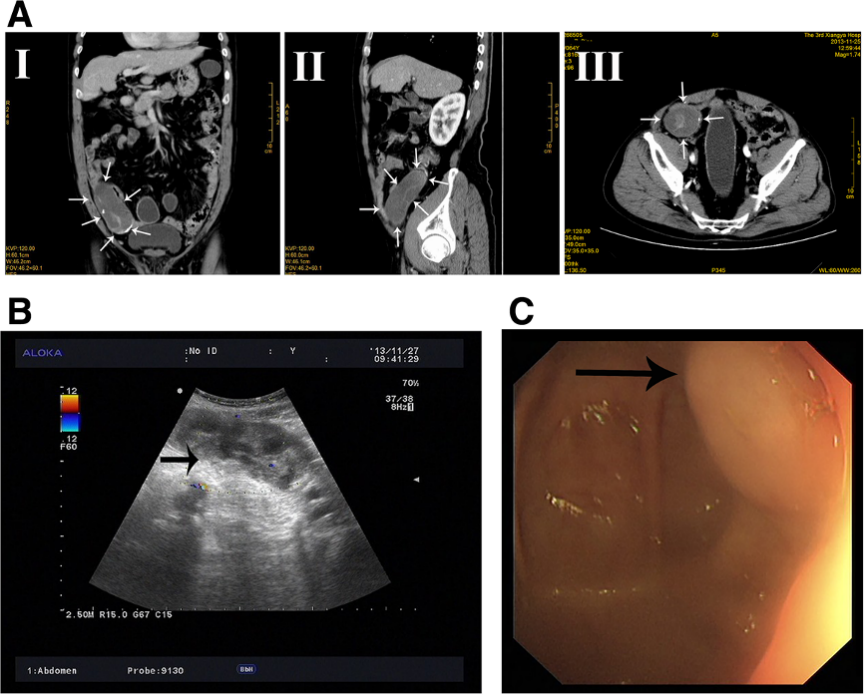
**Preoperative image of the mucinous cystadenoma. (A)** Computed tomography revealed a tumor (arrows). I, coronal imaging; II, sagittal imaging; III, axial imaging. **(B)** Ultrasonography showed distension of the appendix (arrow) that did not compress; the lack of increased Doppler flow in the wall supported a noninflammatory diagnosis. **(C)** Colonoscopy: submucosal tumor-like elevations of the appendiceal orifice (arrow).

**Figure 2:**
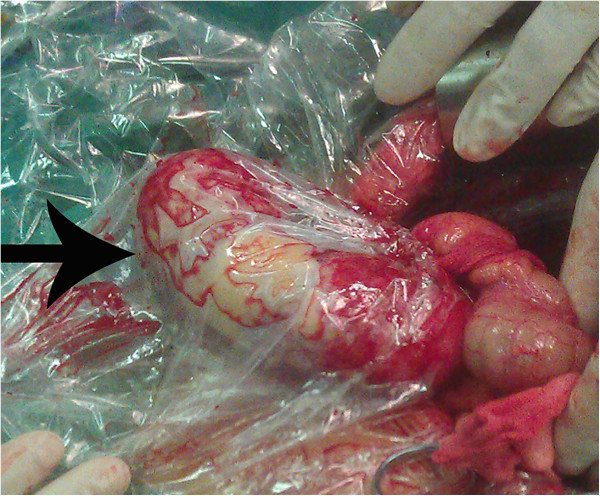
**Intraoperative finding of giant appendiceal mucocele.** Note the plastic bag that was placed on the tumor to prevent epithelial cells from escaping into the peritoneal cavity (arrow).

**Figure 3:**
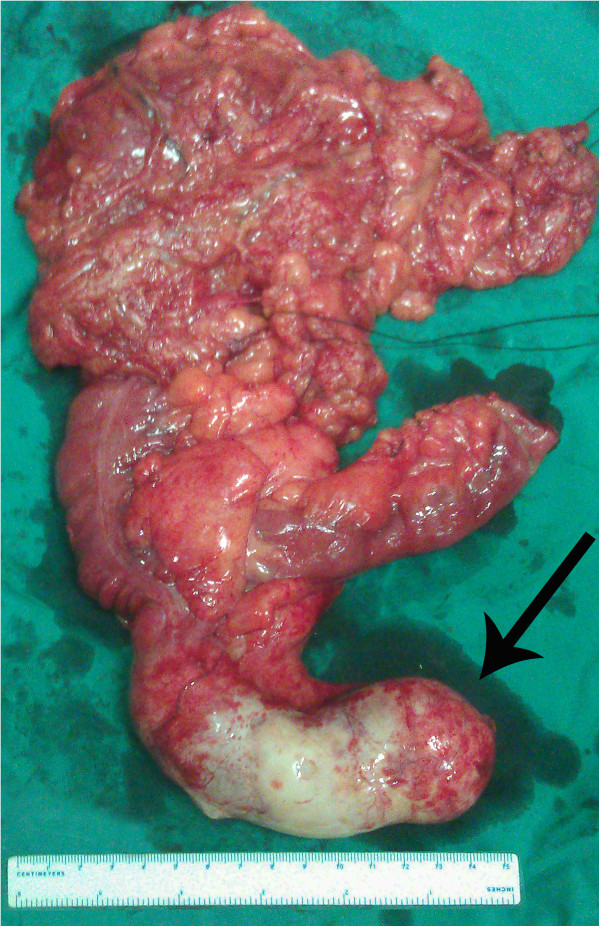
**Resected specimen.** Resected specimen showing giant mucinous cystadenoma of the appendix (arrow).

**Figure 4:**
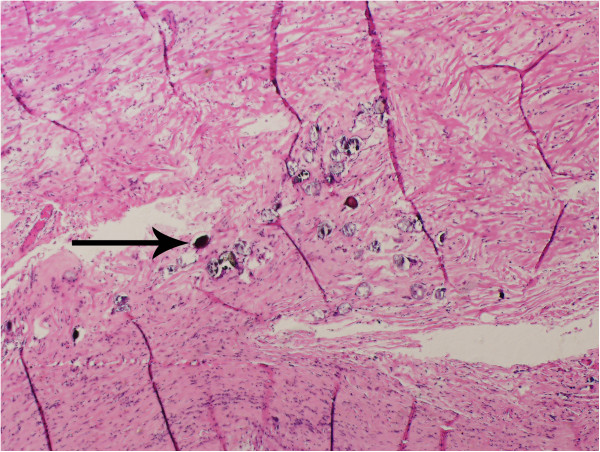
**Pathological findings.** Ap pendiceal mucosa containing Schistosome eggs (arrow).

The patient was a 64-year-old man who was born in Yueyang City, Hunan Province, in Southern China, one of the most heavily endemic areas with schistosomiasis over the past five decades. The patient was admitted to our department with a one-year history of intermittent pain in the lower right quadrant of the abdomen. When the lower right quadrant of the abdomen was palpated, the patient felt pain, but his muscles were not rigid and there was no rebound tenderness. On physical examination, there was no palpable mass in the right lower abdomen. No liver or spleen enlargement was noted. Abdominal ultrasonography revealed a well-encapsulated hypoechoic process measuring 103 × 43 mm (Figure 1B). Computed tomography of the abdomen showed a large, hypodense, tubular, cystic structure measuring 96 × 49 × 48 mm. Moreover, the mass extended below the inferior wall of the cecum (Figure 1A). Colonoscopy revealed a submucosal elevation on the appendiceal orifice (Figure 1C). The patient was diagnosed with mucocele of the appendix, and open surgery was performed. During surgery, a plastic bag was placed on the tumor to prevent epithelial cells from escaping into the peritoneal cavity (Figure 2). At the time of surgery, intraoperative frozen section analysis could not exclude the possibility of malignancy, and the appendiceal base was large. Therefore, right hemicolectomy was performed. The resected right hemicolectomy specimen revealed a dilated appendix, 11 cm in length and 6 cm in diameter, and showed thin fibrous tissue that was distended with intraluminal thick, mucinous material (Figure 3). The final pathological diagnosis was mucinous cystadenoma of the appendix. Moreover, histologic examination revealed numerous calcified, nonoperculated Schistosome eggs within the mucosa, submucosa of the appendix, small intestine, colon, and lymph nodes, associated with surrounding fibrosis (Figure 4). Given the absence of dysplasia in the appendiceal mucosa, we deduced that the mucocele had developed because of fibrosis formed as a result of the obstruction by the Schistosome eggs to the appendiceal outflow of mucin. The patient did not undergo chemotherapy following surgery, recovered uneventfully, and has remained well.

## Discussion

A mucocele of the appendix is a descriptive term that refers to dilation of a lumen because of accumulation of a large amount of mucin. It is a rare lesion in all resected appendiceal tumors. Schistosomiasis of the appendix is also a rare lesion and is observed in 0.4% of all appendectomies performed [7]. Simultaneous giant mucinous cystadenoma of the appendix and intestinal schistosomiasis is extremely rare. To our knowledge, this is the first case ever to be reported in the world.

The characteristic pathological tissue response of appendiceal schistosomiasis is believed to be a granulomatous inflammatory reaction to the schistosomal ova. There is formation of epithelioid cell granulomas, predominantly in the submucosa and serosa, which ultimately undergo fibrosis. The final pathologic examination of the present case revealed calcified Schistosome eggs within the mucosa, submucosa of the appendix, small intestine, colon, and lymph nodes. Considering that mucoceles are caused by mucus distention secondary to an obstruction of the appendix, we speculate that the pathogenesis of appendiceal mucinous cystadenoma in our case is granulomatous inflammation and fibrosis reaction to the Schistosome eggs causing luminal obstruction, finally resulting in intraluminal accumulation of mucoid material.

Mucoceles may be caused by one of four processes: cyst retention, mucosal hyperplasia, mucinous cystadenoma, or mucinous cystadenocarcinoma. Our patient belongs to the mucinous cystadenoma group. Mucinous cystadenoma is a benign tumor characterized by marked distention of the lumen, of up to 6 cm.

The clinical presentation of appendiceal mucoceles is not specific. Very large lesions (up to 51%) will be asymptomatic [8]. A study of Stocchi *et al.* [9] showed that abdominal pain is the most common presentation (27%), followed by abdominal mass (14%), loss of weight (13%), nausea, vomiting, or both (9%), and acute appendicitis (8%). Our patient complained of having had a pain in the right lower quadrant of the abdomen for about one year.

Preoperative diagnosis is difficult but is very important for the selection of an adequate surgical method. Computed tomography, ultrasonography, and colonoscopy are useful for preoperative diagnostics. Computed tomography is the most accurate method in the diagnosis of a mucocele: a typical finding of an appendiceal mucocele is a well-encapsulated cystic mass 2 to 20 cm in diameter communicating with the cecum. Ultrasonography can be used to differentiate between a mucocele and acute appendicitis. For patients with acute abdominal pain in the right lower quadrant of the abdomen, ultrasonography is the first-line diagnostic method. For mucoceles, ultrasonography reveals a sausage-shaped cystic structure in the region of the appendix. Colonoscopy may show elevation of the appendiceal orifice. In some cases, colonoscopy may show the ‘volcano sign’; the appearance of the appendiceal orifice at the center of the mound [10]. Colonoscopy also helps to distinguish between a mucocele and colon cancer.

Treatment for appendiceal mucoceles mainly relies on surgery and partly requires chemotherapeutic washing of the abdomen [8]. One of the cardinal principles of surgical treatment of appendiceal mucoceles is that intact mucoceles do not present future risk for the patient. If the mucocele has ruptured, and epithelial cells escape into the peritoneal cavity, the patient could develop mucinous tumors on peritoneal surfaces and have a very poor prognosis. Therefore, we placed a plastic bag on the tumor to prevent epithelial cells from escaping into the peritoneal cavity. The selection of an adequate surgical method is related to the dimensions and histology of the mucocele. For patients with simple or benign neoplastic mucoceles, appendectomy is adequate when the appendiceal base is intact. For cystoadenoma with a large base, cecal resection is feasible. Right hemicolectomy is recommended when a malignant cause is suspected based on imaging or intraoperative frozen section [11]. In our patient, intraoperative frozen section analysis could not exclude the possibility of malignancy. Additionally, the base of the cystoadenoma was large. Finally, we performed right hemicolectomy.

## Conclusion

We report a rare case of simultaneous giant mucinous cystadenoma of the appendix and intestinal schistosomiasis and provide a brief review of the literature.

## Consent

Written informed consent was obtained from the patient for publication of this case report and accompanying images. A copy of the written consent is available for review by the editor-in-chief of this journal.
